# Supplementary material for: Wound Infection of Snakebite from Venomous Protobothrops mucrosquamatus, Viridovipera stejnegeri and Naja atra in Taiwan: Validation of BITE and Cobra BITE Scoring Systems and their Bacteriological Differences in Wound Cultures
Source: Toxins (Basel). 2023 Jan 15;15(1):78. doi: 10.3390/toxins15010078 (PMC9861491; doi:10.3390/toxins15010078)
Supplement: Supplementary file 1 [file toxins-15-00078-s001.zip › toxins-2119658-supplementary.pdf]

Supplementary Materials: Wound Infection of Snakebite from Venomous *Protobothrops mucrosquamatus, Viridovipera stejnegeri* and *Naja atra* in Taiwan: Validation of BITE and Cobra BITE Scoring Systems and their Bacteriological Differences in Wound Cultures

Heng Yeh, Shi-Ying Gao and Chih-Chuan Lin

Table S1. The characteristics of the validation cohort of the BITE study.

	Wound infection	**No Wound Infection**	*p*-value
	N=35	N=60	
**Demographic characteristics**			
Age	55.71(17.42)	61.00(14.97)	0.12
Male*	24(68.57)	40(66.67)	1
**Laboratory variables**			
WBC(1000/μL)	9.78(4.00)	8.27(5.37)	0.16
Segment	69.26(14.90)	63.29(14.01)	*0.06*
Lymphocyte	22.28(12.40)	28.37(12.83)	*0.03*
Neutrophile lymphocyte ratio	5.04(4.74)	3.52(3.63)	*0.08*
WBC × NLR	56.75(65.33)	35.40(53.93)	*0.09*
HB	14.14(1.40)	14.14(1.43)	0.99
RDW	13.16(1.09)	13.01(0.94)	0.50
PLT (1000/μL)	234.62(62.01)	225.88(64.12)	0.52
Prothrombin time	10.77(1.03)	10.73(1.36)	0.89
aPTT	27.20(1.77)	27.99(2.19)	*0.08*
Cr (mg/dL)	1.05(0.81)	0.94(0.24)	0.43
BUN (mg/dL)	19.03(14.46)	16.59(5.85)	0.46
ALT/GPT (U/L)	40.61(76.27)	21.05(10.64)	0.23
AST /GOT	27.27(12.07)	26.46(7.49)	0.84
Creatine_kinase	217.63(175.16)	244.62(223.13)	0.72
myoglobin	115.11(289.60)	40.87(23.51)	0.37
K (mEq/L)	3.82(0.44)	3.80(0.28)	0.82
Na (mEq/L)	138.55(2.06)	139.34(2.92)	0.15
Glu (mg/ dL)	117.07(52.91)	126.68(70.40)	0.64
**Treatment modalities**			
Antivenin, vial, Median(IQR) (FH does)	2(2-4)	2(2-4)	0.88
**Hospitalization**			
hospital admission, n *	30(85.71)	21(35)	*<0.01*
length of hospitalization	5.31(4.40)	1.79(2.85)	*<0.01*

*X^2 test

Table S2. The characteristics of the validation set of the Cobra Bite study.

	Wound infection	No wound infection	p-value
	N=11	N=14	
**Demographic characteristics**			
Age	57.82(22.81)	55.79(17.36)	0.80
Male	7(63.64)	13(92.86)	0.99
**Laboratory variables**			
WBC(1000/μL)	7.83(2.82)	7.84(3.22)	0.99
Segment	65.15(12.76)	55.79(12.77)	*0.08*
Lymphocyte	26.22(10.82)	34.65(13.27)	*0.10*
Neutrophile lymphocyte ratio	3.58(3.29)	2(1.17)	0.16
WBC × NLR	33.71(43.81)	15.72(11.75)	0.21
HB	14.46(2.04)	15.29(2.44)	0.38
RDW	13.15(0.90)	12.56(1.30)	0.21
PLT (1000/μL)	249.18(92.94)	235.86(53.34)	0.66
Prothrombin time	10.59(0.71)	10.61(0.47)	0.95
aPTT	27.94(3.61)	27.42(2.69)	0.69
Cr (mg/dL)	0.95(0.18)	0.89(0.21)	0.47
BUN (mg/dL)	16.54(4.87)	13.95(4.20)	0.21
ALT/GPT (U/L)	31.22(15.55)	39.29(31.92)	0.43
AST /GOT	32.50(8.17)	34.92(18.65)	0.77
Creatine_kinase	242.14(213.80)	175.67(101.21)	0.46
myoglobin	44.35(18.46)	45.21(45.38)	0.97
K (mEq/L)	3.77(0.26)	3.71(0.30)	0.64
Na (mEq/L)	140.11(2.89)	138.57(1.95)	0.14
Glu (mg/ dL)	141.67(70.06)	159.71(82.45)	0.68
**Treatment modalities**			
Antivenin, vial, Median(IQR) (FN dose)	6(2-10)	4(2-6)	0.23
**Hospitalization**			
hospital admission, n	10(90.91)	7(50)	*0.04*
length of hospitalization	6.27(4.38)	1.64(1.86)	*0.01*

Table S3. bacteriology culture reports and antibiotic susceptibility.

Naja atra bites	**AMP**	**AMK**	**CAZ**	**CIP**	**SCF**	**CRO**	**CXM**	**CFZ**	**ETP**	**GEN**	**LVX**	**PEN**	**SAM**	**TZP**	**TEC**	**VAN**
Morganella morganii		S	S	S	S	S	R	R	S	S	S		R	S		
Proteus vulgaris		S	S	S	S	S	R	R	S	S	S		S	S		
E.faecalis	S									S(120ug)		S			S	S
Morganella morganii		S	S	S	S	S	R	R	S	S	S		S	S		
Proteus vulgaris		S	S	S	S	S	R	R	S	S	S		S	S		
Enterococcus faecalis	S									S(120ug)		S			S	S
Proteus vulgaris		S	S	S	S	S	R	R	S	S	S		S	S		
Enterococcus faecalis	S									S(high dose)		S			S	S
Enterobacteriaceae		S	S	S	S	S	S	R	S	S	S		S	S		
Kleb.pneumoniae		S	S	S	S	S	S	S	S	S	S		S	S		
E. faecalis	S									S(high dose)		S			S	S
Protobothrops mucrosquamatus, Viridovipera stejnegeri bites	**AMP**	**AMK**	**CAZ**	**CIP**	**SCF**	**CRO**	**CXM**	**CFZ**	**ETP**	**GEN**	**LVX**	**PEN**	**SAM**	**TZP**	**TEC**	**VAN**
Enterobacter cloacae complex		S	S	S	S	S	R	R	S	S	S		R	S		
Coagulase(-) *staphylococcus*																

Abbreviations: PEN, penicillin; PIP, piperacillin; SAM, ampicillin-sulbactam; AMP, ampicillin; GEN, gentamicin; TEC, teicoplanin; VAN, vancomycin; AMK, amikacin; CIP, ciprofloxacin; CFZ, cefazolin; CXM, cefuroxime; CRO, ceftriaxone; CAZ, ceftazidime; SCF, cefoperazone-sulbactam; ETP, ertapenem; LVX, levofloxacin; TZP, piperacillin-tazobactam.

Table S4. Abbreviation table.

Abbreviation	Definition
ALT/GPT	alanine aminotransferase/ glutamate pyruvate transaminase
aPTT	activated partial thromboplastin time
AST/GOT	aspartate aminotransferase/glutamic oxaloacetic transaminase
AUC	area under the curve
BITE study	Bacteriology of Infections in Taiwanese snake Envenomation study
BUN	blood urea nitrogen
CGMH	Chang Gung Memorial Hospital
CI	confidence interval
Cobra BITE study	Cobra Bacteriology of Infections in Taiwanese snake Envenomation study
Cr	blood creatinine
ED	emergency department
FH	freeze-dried hemorrhagic
FN	freeze-dried neurotoxic
Glu	blood glucose
Hb	hemoglobin

ICU	intensive care unit
IV	intravascular
IQR	interquartile range
K	potassium
Na	sodium
NLR	neutrophil-lymphocyte ratio
PLT	platelet count
RDW	red blood cell distribution width
ROC	receiver operating characteristic
SD	standard deviation
WBC	white blood cell count
